# Heart Failure in Sub-Saharan Africa

**DOI:** 10.2174/1573403X11309020008

**Published:** 2013-05

**Authors:** Gerald S Bloomfield, Felix A Barasa, Jacob A Doll, Eric J Velazquez

**Affiliations:** 1 Division of Cardiovascular Medicine, Duke University, Durham, NC; 2 Duke Clinical Research Institute, Duke University, Durham, NC; 3 Department of Medicine, Moi Teaching and Referral Hospital, Eldoret, Kenya

**Keywords:** Heart failure, sub-Saharan Africa.

## Abstract

The heart failure syndrome has been recognized as a significant contributor to cardiovascular disease burden in sub-Saharan African for many decades. Seminal knowledge regarding heart failure in the region came from case reports and case series of the early 20^th^ century which identified infectious, nutritional and idiopathic causes as the most common. With increasing urbanization, changes in lifestyle habits, and ageing of the population, the spectrum of causes of HF has also expanded resulting in a significant burden of both communicable and non-communicable etiologies. Heart failure in sub-Saharan Africa is notable for the range of etiologies that concurrently exist as well as the healthcare environment marked by limited resources, weak national healthcare systems and a paucity of national level data on disease trends. With the recent publication of the first and largest multinational prospective registry of acute heart failure in sub-Saharan Africa, it is timely to review the state of knowledge to date and describe the myriad forms of heart failure in the region. This review discusses several forms of heart failure that are common in sub-Saharan Africa (e.g., rheumatic heart disease, hypertensive heart disease, pericardial disease, various dilated cardiomyopathies, HIV cardiomyopathy, hypertrophic cardiomyopathy, endomyocardial fibrosis, ischemic heart disease, cor pulmonale) and presents each form with regard to epidemiology, natural history, clinical characteristics, diagnostic considerations and therapies. Areas and approaches to fill the remaining gaps in knowledge are also offered herein highlighting the need for research that is driven by regional disease burden and needs.

## INTRODUCTION

Heart failure (HF) is a major public health problem worldwide. The available data suggest that while the morbidity due to HF is great in many parts of the world, the etiologies differ [1]. The most common underlying cause of HF in high-income countries is coronary artery disease [2]. In sub-Saharan Africa (SSA), the predominant causes have traditionally been ascribed to rheumatic heart disease, hypertensive heart disease and cardiomyopathy [3-5]. Recent data from the sub-Saharan African Survey of Heart Failure (THESUS-HF) underscore the significant contribution of hypertension [6]. Coronary artery disease was once a rarity on the African continent [7, 8] leading some to posit immunity to coronary atherosclerosis among black Africans well into the 1970s [9, 10]. Newer data confirm the beginnings of an epidemiologic transition to more degenerative forms of HF [11]. Other forms of HF which are not unique to SSA but are commonly found include human immunodeficiency virus (HIV) associated cardiomyopathy, tuberculous pericardial disease, cor pulmonale and peripartum cardiomyopathy among other causes. 

Factors unique to developing regions such as SSA make HF an especially challenging condition. Compared to other parts of the world, HF in SSA tends to occur at a much younger age. This finding could be due to the major contribution of rheumatic heart disease or early presentation of other degenerative causes. Misdiagnosis or under-diagnosis of HF is likely to occur where access to echocardiography or serologic markers is poor [12]. While HF with systolic dysfunction appears to be the most common form [13], most of the literature in this area occurred prior to the recognition of HF with preserved ejection fraction. Treatment patterns suggest that evidence-based medications are underutilized [14] and compliance is poor [13]. The hospital case-fatality rate among those with HF ranges from 9-12.5% [15].

This review is designed to summarize what is known about the current forms of HF in SSA and is timely given the high prevalence of HF, growing interest and investment into cardiac disease research on the continent [16, 17] and ongoing shifts in epidemiology[18]. Herein, we discuss several forms of HF that are commonly encountered in SSA and present what is currently known about disease epidemiology, natural history, clinical characteristics, diagnostic considerations and therapies. It is important to note that, unlike the high-income regions where the epidemiology of HF has been well described, the bulk of the literature on HF in SSA is derived from hospital-based studies [5]. By offering this comprehensive review, we aim to condense the body of literature on this topic into a resource for students, clinicians, researchers and policy makers who are interested in and/or involved in the care of patients with HF on the African continent.

## RHEUMATIC HEART DISEASE 

### Description and Pathophysiology

Valvular heart disease in SSA is almost always due to sequelae of an infectious disease rather than degenerative changes. Recurrent pharyngeal infections with group A β-hemolytic streptococci and subsequent acute rheumatic carditis predispose to the development of rheumatic heart disease (RHD) - a chronic progressive condition with no known medical therapy. Valvular thickening eventually impairs function with subsequent valvular regurgitation. Over time, valvular stenosis may begin to predominate with further restriction in leaflet mobility and development of a transvalvular pressure gradient. Pure mitral regurgitation is the most common form of RHD in those younger than 20 years (>70% of cases) while mitral stenosis and mixed valvular disease becomes more common with advancing age [19]. Pulmonary edema, arrhythmias, HF and eventual death are the ultimate result without corrective therapy.

### Epidemiology

While SSA is home to only 10% of the world’s population, nearly 50% of the 2.4 million children affected by RHD reside here [20]. School-based studies of children using clinical criteria alone reveal RHD prevalence rates between 2.7 per 1000 (Kenya) and 14.3 per 1000 (Democratic Republic of the Congo) [21, 22]. Importantly, when echocardiographic screening criteria are used to define RHD, the prevalence is up to ten times higher than using clinical criteria alone [23-25]. In one study in western Kenya, newly diagnosed RHD was found in 41% of patients referred for an echocardiogram for HF symptoms [26].

The classic natural history studies of Rowe *et al*. [27], Oleson [28] and Grant [29] have shown that while the overall rate of clinical progression of rheumatic mitral stenosis is low, there is considerable variation between patients. These studies from the United States, Denmark and England, respectively, demonstrate a long lag time (>20-40 years) before the majority of patients with rheumatic valvular involvement progress to severe disease. The progression rate among patients in SSA is largely unknown, however, the disease appears to be more malignant here. Oli and Asmera reviewed the deaths of 115 patients with RHD from Ethiopia [30]. The median age of death was less than 25 years with HF being the most common cause. Symptoms of mitral stenosis also begin earlier in life and may be present in children who are less than five years of age [31]. Bacterial endocarditis is a dreaded complication of RHD requiring attention to prophylaxis in selected patients [19]. It is widely believed that the majority (70% in some reports) [30] of patients will die at a young age without surgical valve repair or replacement. Lack of access to surgery contributes to the 1.4 million deaths per year attributed to complications of RHD [32].

### Clinical Characteristics

Rapid progression and symptomatic presentation of RHD is the rule more than the exception in SSA and both sexes are affected to a similar degree [23, 24]. Even with severe mitral regurgitation, many patients will remain asymptomatic at rest until there is left ventricular failure, pulmonary hypertension or the onset of atrial fibrillation. The most common symptoms are exertional dyspnea and fatigue. Younger children with significant mitral regurgitation may be mistaken for having common childhood illnesses such as gastroenteritis, reflux or asthma. Older children may also present with exercise intolerance, wheezing or palpitations. With predominantly stenotic mitral valve lesions, symptoms are related to a high pressure gradient between the left atrium and ventricle and usually do not develop until the effective mitral valve orifice falls below 2cm^2^. Arterial embolism as a presenting characteristic (i.e., cerebrovascular or peripheral arterial occlusion) is rare and its presence does not correlate with the severity of mitral valve stenosis but is related to the presence of atrial fibrillation [33]. Patients with moderate to severe mitral regurgitation will have a displaced apex to the anterior or mid-axillary line and a loud pansystolic murmur at the apex. A diastolic murmur of mitral stenosis or a mid-diastolic murmur of increased transmitral flow may be heard. 

### Diagnostic Considerations

The most recent guidelines for diagnosing RHD recommend using echocardiography and incorporating morphologic criteria as well as Doppler-based assessment of regurgitant flow. Presumably, these expanded criteria offer the possibility of correctly identifying more subclinical disease in time to prevent progression to clinical RHD but there is no empiric evidence to support this assumption. The World Heart Federation guidelines employ two-dimensional, continuous wave and color Doppler echocardiography to categorize findings into three categories - definite RHD, borderline RHD and normal – with different criteria for those above and below 20 years of age [34]. With subcategorization based on type of valvular involvement, a total of eight categories exist. What remains unknown is the impact of discovering subclinical disease and the practicalities of implementing echocardiographic-based screening programs in the poorest regions of the world where RHD has the greatest burden and access to interventions are limited or unavailable.

### Therapy

There are no proven medical therapies to alter the natural history of RHD [35, 36]. Medical therapy is largely preventive or prophylactic for complications. South African guidelines suggest that in communities where rheumatic fever is endemic, all cases of sore throat among children between 3 and 15 years of age should be regarded and treated as streptococcal infection unless there are signs suggesting otherwise (i.e., oral ulceration, hoarseness, watery nasal discharge and/or conjunctivitis) [32]. Secondary prevention of rheumatic fever aims to limit the number of recurrent infections and presumably decrease the likelihood of progression to RHD. The latter has never been shown empirically [37]. Intramuscular penicillin should be administered every 2-4 weeks according to international recommendations [22]. Adjunctive medical therapy may also include antiarrhythmic, diuretic or anticoagulation therapy when indicated. Definitive therapy of symptomatic RHD will involve valvuloplasty as a temporizing measure before surgical repair or replacement of the involved valve(s). In developing countries, the large number of surgical procedures on young persons with RHD has been described as “attempting to mop up the water on the floor while leaving the faucet open” [38], which testifies to the unfinished work of vaccine development and primary prevention. 

## HYPERTENSIVE HEART DISEASE

### Description and Pathophysiology

Hypertensive heart disease is the cardiac damage related to chronic systemic arterial hypertension. Nowadays, among those >65 years of age, the prevalence of hypertension is roughly 30-40% in rural West Africa, 50% in semi-urban West Africa [39], 50-60% in South Africa [40] and 30-50% in East Africa [41, 42]. Taking the region as a whole using single-center reports and a systematic review, Ibrahim and Damasceno estimate that the prevalence of hypertension (blood pressure >160/95 mmHg) in adults >=55 years increased from 54 to 78% between 1998 and 2003 [43]. Based on ageing of the population and adoption of Western lifestyles, the African Union estimates that 10-20 million people in SSA may currently be affected by hypertension earning it the moniker -“the greatest health challenge after AIDS” [44]. 

The causes of hypertensive heart disease in SSA seem to be similar to the rest of the world. A number of genes have been implicated in the development of cardiomyocyte hypertrophy in patients with essential hypertension which affect intracellular signaling, degradation of normal extracellular collagens and contractile dysfunction among other functions ultimately leading to left ventricular hypertrophy and HF [45]. As opposed to these genetic variants being deterministic, there is likely a “gene-environment interaction” such as seen in black Americans whereby weight gain, high salt intake and psychosocial factors may facilitate the rapid development of hypertension and hypertensive heart disease in susceptible individuals [46].

### Epidemiology

In SSA, hypertensive heart disease consistently ranks in the top three causes of HF from all regions of the continent from the 1950s to the present (Table 1). Varying definitions of hypertensive HF have been employed during this time frame and limits direct comparison between studies. The diagnostic criteria commonly include symptoms of heart failure, having a documented high blood pressure and evidence of left ventricular hypertrophy (by electro- or echocardiogram). It is important to note however that although high blood pressure is very common among Africans with a diagnosis of HF (60-80% prevalence) [6, 18, 66] progression to systolic failure and ventricular dilatation is less common than the development of high end-diastolic pressure and diastolic dysfunction [67].

### Clinical Characteristics

The clinical presentation of patients with hypertensive HF is similar to other patients with HF with the caveat that symptoms of diastolic dysfunction and elevated ventricular filling pressures (in contrast to low cardiac output) may be present earlier on in the course. When left ventricular hypertrophy is present the classic physical findings are an abnormally sustained apical impulse that is enlarged and displaced outside of the midclavicular line. An S4 gallop heard during auscultation is common in chronic hypertension and reflects the decreased elasticity of a hypertrophied ventricle during late diastole (i.e., during atrial contraction). 

### Diagnostic Considerations

The two most common tools used in SSA to define left ventricular hypertrophy are the electrocardiogram and the echocardiogram. The former provides information on voltage, cardiac rhythm, electrolyte imbalances, PR interval and QT interval while the echocardiogram will also provide determination of wall thickness, atrial size, left and right ventricular function and hemodynamics. The sensitivity and specificity of the electrocardiogram for left ventricular hypertrophy are approximately 7-74% and 41-98%, respectively, and no single criteria has the highest sensitivity, specificity, accuracy or correlation with cardiac magnetic resonance estimated left ventricular mass index [68-71]. Echocardiography is the most commonly used modality to identify, quantify and monitor the progression of left ventricular hypertrophy owing to its portability, reproducibility and correlation with left ventricular mass at necropsy [72]. The role of biomarkers in diagnosing hypertensive HF is still being defined and does not yet impact treatment decisions [73].

### Therapy

Treatment for hypertensive HF follows the guidelines for the management of patients with HF according to clinical stage [74]. Antihypertensive therapy has been shown in over 400 clinical studies to promote regression of left ventricular mass [75]. In addition, therapies commonly prescribed to reduce blood pressure [e.g., angiotensin converting enzyme inhibitors, angiotensin receptor blockers) decrease mortality from HF [76]. For these reasons, a patient with HF and hypertension should be treated initially with anti-hypertensive medications that could potentially reduce the morbidity and mortality of both conditions. Diuretic-based antihypertensive therapy has repeatedly been shown to prevent HF in a wide range of target populations [77]. Angiotensin converting enzyme inhibitors and beta- blockers are also effective in the prevention of HF [78], whereas calcium channel blockers and alpha receptor antagonists are less effective in preventing the HF syndrome [79]. When blood pressure is acutely elevated in a patient with HF, accurate control of blood pressure is crucial but lowering the blood pressure too low and too quickly is associated with poor outcome [80]. 

## PERICARDIAL DISEASE

### Description and Pathophysiology

Clinically, two syndromes of pericardial diseases predominate: pericarditis and pericardial effusion. Tuberculosis is by far the commonest cause of pericardial inflammatory disease in SSA, accounting for about 10% of patients hospitalized with HF [59]. Mycobacterium reach the pericardium via three main routes: retrograde from mediastinal, peritracheal and peribronchial lymph nodes, hematogenous spread during tuberculosis bacteremia and by direct spread from the lungs and pleura. In immune-competent hosts, spread is predominantly via lymphatics and the condition is frequently pauci-bacillary in which the morbidity is related to the ferocity of the immune response and not to the virulence of the pathogen itself [81]. On the contrary, hematogenous spread is the commonest route in HIV disease [82]. Large effusions and hemodynamic compromise are common, and constriction occurs as a late complication. Other infectious causes such as bacterium, fungi and parasites are less common [83].

Others important causes of pericardial disease in SSA include metastatic neoplastic (e.g., Kaposi’s sarcoma) and secondary metastatic tumors (e.g., lung and breast cancer, lymphoma)[84]. Pericarditis due to hypothyroidism, uremia, connective tissue diseases, radiation injury, Dressler’s syndrome, drugs and trauma occur in SSA but their contribution to morbidity is unknown.

### Epidemiology

In one South African center, tuberculosis accounted for 70% (162 of 233) of all cases referred for diagnostic pericardiocentesis [85]. Most HIV seropositive patients had tuberculous pericardial effusions. The natural history of pericardial effusion depends on the underlying etiology, being generally morbid in patients with neoplastic pericardial effusion and tuberculous pericarditis [86, 87]. In patients with tuberculous pericarditis, HIV co-infection worsens the prognosis (mortality rates 7% in HIV seronegative patients compared to 40% in HIV seropositive patients) [86]. 

### Clinical Characteristics

Classic symptoms of pericarditis include chest pain that typically radiates to both trapezius muscular ridges. Patients may also complain of a viral prodrome of fever, non-productive cough, myalgias and malaise. On physical examination, typical findings include a pericardial friction rub early in the course which may subsequently disappear when a pericardial effusion develops. A pericardial knock usually signifies constriction. In the presence of significant effusion, jugular venous pressure is increased, with prominent systolic x descent and blunted diastolic y descent. The precordium becomes quiet and blood pressure falls. The classic Beck’s triad of “surgical tamponade” is rarely seen in the medical patients [88]. With reduction in cardiac output, pulse pressure is narrow and systemic venous congestion causes hepatomegaly, peripheral edema, and ascites.

### Diagnostic Considerations

Where resources are limited, a chest X-ray (CXR) may often be the only imaging modality available to the clinician. *Reuter et al.* showed that CXR positively identified 53% (n = 90) of patients with pericardial effusion [89]. All of them had an enlarged cardiac silhouette and in the majority of cases, the cardiac shadow was globular with distinct margins. The amount of fluid drained correlated with the radiographic finding of cardiac enlargement in this study.

The ECG shows ST-segment elevation (in acute pericarditis) that is different from that in acute myocardial infarction: being more diffuse, involving both limb and precordial leads. After several days, the ST segment returns to baseline in a predictable manner [90]. Other ECG findings include PR segment depression and electrical alternans. Echocardiography remains the most sensitive tool for the diagnosis of pericardial effusion by showing an echo-free space around the heart [83]. In addition to measuring the size of the effusion, echocardiography allows for an assessment of any hemodynamic consequences. The absence of a pericardial effusion on echocardiography does not exclude the diagnosis of acute pericarditis.

Diagnostic pericardiocentesis yields a specific diagnosis in only 7% of cases of pericardial effusion without tamponade [91]. The pericardial fluid is blood stained in 80% of cases of tuberculous pericarditis [92]. However, because malignant disease and the late effects of penetrating trauma may also cause bloody pericardial effusion, confirmatory testing for tuberculosis is important [93]. Tuberculous pericardial effusions are typically exudative and characterized by a high protein content and increased leukocyte count, with a predominance of lymphocytes and monocytes. The diagnostic yield of pericardial biopsy without pericardioscopy is low [94]. Overall, the etiology is determined in only about a quarter of patients [83]. A scoring system, the Tygerberg score, has been validated for tuberculous pericarditis and is currently being utilized in research settings [95]. One point each is allotted for the presence of weight loss, night sweats or fever. Two points are given for fever and three points for either serum globulin >40g/L or blood leukocyte count <10 x 10^9. A score of 6 or greater is highly suggestive of tuberculous pericarditis.

### Therapy

Treatment regimens recommended for pericardial TB are the same as for pulmonary tuberculosis, consisting of 6 months of anti-tuberculous antibiotics. Corticosteroids are frequently prescribed for tuberculosis pericarditis but the evidence to support their use is limited. The effectiveness of systemic steroids is currently under investigation in a large multi-centre trial to evaluate their role (IMPI Trial) [95]. Non-steroid anti-inflammatory drugs remain the cornerstone of treatment of other forms of pericarditis and colchicine reduces the recurrence rate [96]. Corticosteriod therapy, on the other hand, favors recurrence if given during the index attack. Therapeutic pericardiocentesis is usually a life-saving intervention and is essential in patients with cardiac tamponade. In chronic pericardial constriction, definitive treatment is surgical pericardial decortication, where both the visceral and parietal pericardium are widely resected [84]. 

## DILATED CARDIOMYOPATHY

### Description and Pathophysiology

Dilated cardiomyopathy (DCM) refers to a heterogeneous group of heart muscle diseases of diverse etiologies that are characterized by dilatation and impaired contraction of the left and/or right ventricles. The histological findings are frequently nonspecific. Presentation is usually with progressive HF. Arrhythmias, thromboembolism, and sudden death are common and may occur at any stage. It is a common cause of HF in SSA [92]. Important causes in SSA include HIV cardiomyopathy, peripartum cardiomyopathy, myocarditis, infiltrative disease (i.e., iron overload), alcohol-induced and familial/genetic forms [20].

Familial disease accounts for one third to one half of cases of DCM [97]. More than 40 genes have been identified with the most common mode of inheritance being autosomal dominant [98]. Studies from South Africa have shown an association with HLA-DR1 and DRw10 antigens as well as a common mitochondrial polymorphism with idiopathic DCM [99]. The genes involved encode components of a wide variety of cellular compartments and pathways. Overall, the diverse changes in cardiomyocyte structure and function result in organelle degradation and apoptosis. No clinical or histological criteria, other than family history and careful examination of relatives, have been derived to distinguish familial from nonfamilial DCM on the basis of phenotype. 

Alcoholic cardiomyopathy is the commonest form of toxin-induced cardiomyopathy. Although the pathogenesis and factors that determine patient susceptibility are still poorly understood, alcohol is believed to be toxic to cardiac myocytes via oxygen free radical damage and defects in cardiac protein synthesis [100]. The risk of developing alcoholic DCM is related to both mean daily alcohol intake and the duration of drinking [101]. In traditional African societies, communal drinking makes it difficult to quantify the amount of alcohol consumed over time and what may be objective is only the type, duration or cost of use. Moreover, traditional brews have diverse alcohol concentrations ranging from almost pure alcohol (e.g., *changaa *in East Africa) to levels equivalent to those of commercial beers. Epidemics of impurities in commercially produced beers producing HF (e.g., arsenic and cobalt beer-drinkers diseases) are for the most part historical [102], however, the extent to which impurities in traditional brews contribute to HF in SSA has not been well investigated. Whereas nutritional deficiencies were once thought to precipitate the myopathy of chronic alcohol ingestion, it is now clear that malnutrition is not the leading factor [103].

Myocarditis is an etiologic factor for DCM in up to 24% of cases and is associated with autoimmune disorders [104]. Auto-immune mediated myocarditis due to inappropriate immunologic responses has been identified as a possible precursor of DCM in SSA [105]. Commonly reported infective agents include toxoplasmosis and coxsackie viruses though this is not a consistent finding in studies where serology is used to diagnose the infections [104, 106].

The cardiomyopathy of iron overloading is an infiltrative disease that does not produce left ventricular hypertrophy. Hereditary hemochromatosis as well as other forms of iron deposition disease are found in SSA [107-109]. A specific form of iron overload (so-called African iron overload) was initially thought to result from ingestion of large amounts of iron from beer brewed in steel drums [110]. However, iron overload cardiomyopathy as such only occurs in a small number of beer drinkers suggesting that a genetic factor is also present which may predispose to iron loading. Phlebotomy performed weekly results in marked improvement of ventricular function and decrease in left ventricular size [111]. 

Peripartum cardiomyopathy refers to an idiopathic form of cardiomyopathy presenting with HF with left ventricular systolic dysfunction towards the end of pregnancy or in the months following delivery. It is a diagnosis of exclusion. It is a rare from of cardiomyopathy in high-income countries (incidence rates 1/2500 – 4000 live births) but fairly endemic in SSA (1/300 in South Africa, 1/100 in Nigeria) [112, 113]. A number of factors have been postulated including traditional cardiovascular risk factors, African descent, maternal age, parity and malnutrition. Recent research from South Africa suggest involvement of a cascade involving oxidative stress, the cathepsin D protease, and prolactin hormone in the pathophysiology of peripartum cardiomyopathy [114]. Pro-apoptotic prolactin subfragments are largely responsible for the myocardial damage and in small pilot studies treatment with modalities that modulate this pathway confer clinical benefit to patients [115]. Genetic susceptibility as well as environmental exposures are the likely ultimately factors determine the development of clinical disease [116]. 

### Clinical Features

Overall, patients with DCM present with typical symptoms and signs of HF including fatigue, dyspnea, orthopnea, and paroxysmal nocturnal dyspnea. Palpitations and arrhythmias may result in syncope. The pulse pressure may be narrow with elevated diastolic pressure caused by systemic vasoconstriction. A displaced apical impulse, and an apical murmur of mitral regurgitation are common. On cardiac auscultation, S_3_ and S_4_ gallops are common especially when decompensated. Fluid retention marked by elevated jugular venous pressure and peripheral edema are typical.

### Diagnostic Considerations

HIV testing is usually indicated in any patient presenting with DCM owing to its high prevalence. Consensus opinion also recommends other diagnostic laboratory studies to include complete blood count, urinalysis, serum electrolytes (including calcium and magnesium), blood urea nitrogen, serum creatinine, fasting blood glucose, lipid profile, liver function tests and thyroid stimulating hormone prior to labeling a patient with idiopathic cardiomyopathy [74]. Serum viral antibody testing are occasionally measured but the yield is low [117]. Other laboratory testing (e.g., for connective tissue diseases or pheochromocytoma) is recommended when these diagnoses are specifically considered. Similarly, endomyocardial biopsy should be considered when a specific diagnosis is suspected that would influence therapy [74]. Echocardiography allows the evaluation of cardiac chamber sizes and wall thickness as well as systolic and diastolic function. It is one of the most important tools to rule out other causes of HF such as valvular heart disease, pericardial effusion or other cardiomyopathies.

### Therapy

Management of HF in patients with DCM follows similar principles as with other forms of HF with diuretics, afterload reduction and adrenergic blockade being the cornerstone. Patients receiving optimal medical therapy but who remain symptomatic should be considered for cardiac defibrillator or cardiac resynchronization therapy if they meet other selection criteria and if these options are available. Advanced therapies such as cardiac transplant or left ventricular assist devices in those with endstage disease may only be available in few countries.

It is noteworthy that in patients with peripartum cardiomyopathy, angiotensin converting enzyme inhibitors, angiotensin receptor blockers and spironolactone should be avoided in the prenatal period because of fetal toxicity. Hydralazine is particularly safe as an afterload reducing agent during this stage and digitalis can be used to increase contractility and for rate control. Bromocriptine has been shown to improve both clinical and cardiac function in pilot clinical trials but has yet to be replicated in larger studies [115]. Subsequent pregnancies are usually discouraged especially in patients whose left ventricular ejection fraction was <25% at the time of diagnosis or has not normalized [118].

Standard treatment of iron overload cardiomyopathy currently includes dietary management, phlebotomy, and chelating agents. The role of calcium channel blockers in limiting iron transport into cardiac myocytes is being explored [119]. In alcoholic cardiomyopathy the benefit of total abstinence has long been recognized [120]. Abstinence for alcoholic cardiomyopathy leads to a better prognosis compared to the idiopathic forms [121].

## HIV CARDIOMYOPATHY

### Description and Pathophysiology

HIV infection is associated with significant cardiovascular morbidity and mortality in SSA. Cardiac disease in HIV infection occurs with and without combined anti-retroviral therapy. In addition to the HIV virus itself traditional cardiovascular factors, the host response to the virus and the effects of anti-retroviral therapy are all thought to contribute to the cardiovascular disease burden [122]. Dilated cardiomyopathy, acute myocarditis, cor pulmonale due to HIV associated pulmonary hypertension and cardiac tumors are also known to occur [123]. HIV viral particles (e.g., glycoprotein 120, Tat and Nef proteins) can directly impair cardiac myocyte contractility by causing myocarditis although the virus itself does not appear to enter receptor-negative myocytes [124, 125]. Auto-immunity has also been described [126]. Reports from the West show that acute myocarditis may also occur as a result of opportunistic infections due to infection with *Toxoplasma gondii,* coxsackievirus group B, cytomegalovirus, adenovirus or Epstein-Barr virus [127]. A study from Kinshasa which reviewed histopathological specimens from 16 patients showed that myocarditis was attributed to *Toxoplasma gondii *in 3 cases, to *Cryptococcus neoformans *in 3, to *Mycobacterium avium intracellulare *in 2 and to direct HIV infection in 8 patients [128].

### Epidemiology

Cardiomyopathy in HIV seropositive patients is associated with low socioeconomic status, a longer duration of HIV infection, low total lymphocyte count, low CD4 count, high HIV-1 viral load and low plasma levels of selenium [123]. A CD4 count <100 appears to be an important threshold below which the risk of developing cardiomyopathy increases in African and non-African populations [129, 130]. Cross-sectional echocardiographic studies from SSA indicated a prevalence of up to 57% in hospitalized patients [131]. Incidence rates were 17% over 18 months in outpatients in Kinshasa [132]. A prospective study of 157 HIV-positive patients in Kinshasa showed that about half of the patients developed a cardiac abnormality over a 7-year period [128]. The mortality due to HIV-associated cardiomyopathy is high, reaching 15-20% among Congolese patients [133].

### Clinical Characteristics

A significant proportion of patients with HIV-associated cardiomyopathy are free of specific cardiovascular signs and symptoms [134]. In the Heart of Soweto study, 24% of patients with HIV-associated cardiomyopathy had asymptomatic left ventricular dysfunction [124] and a study from Zimbabwe showed that the majority of patients presented with asymptomatic left ventricular dysfunction [135]. HIV-associated cardiomyopathy is associated with rapid progression to death within 100 days of diagnosis in patients who are not treated with antiretroviral therapy [130] .

### Therapy

Treatment of cardiomyopathy associated with HIV follows guidelines for standard HF therapy [74]. There is no prospective evidence to suggest that combined anti-retroviral therapy has a beneficial effect on HIV-associated cardiomyopathy but it is usually instituted because presence of cardiomyopathy is currently recognized as HIV stage 4 disease [136]. A retrospective study from Italy, however, has shown that patients on combined anti-retroviral therapy have a lower incidence of cardiomyopathy compared to single-drug regimens [137]. 

## HYPERTROPHIC CARDIOMYOPATHY

### Description and Pathophysiology

Hypertrophic cardiomyopathy (HCM) is the most common genetic cardiovascular disease [138]. Autosomal dominant genetic mutations resulting in HCM primarily involve the cardiac sarcomere but may also involve the connective tissue matrix. Currently, it is known that many cases of sporadic and familial HCM are caused by more than 150 distinct mutations in at least nine different genes [139]. In South Africa, 3 distinct founder mutations have been found in about a half of the patients with HCM [140]. These mutations are enriched in South Africa owing to the sub-population history and degree of European ancestry [140]. One of these mutations (troponin T R92W) is associated with early sudden cardiac death often with minimal hypertrophy and is found especially in young males in South Africa [141].

HCM is characterized by left ventricular hypertrophy of various morphologies, with a wide array of clinical manifestations and hemodynamic abnormalities. Depending upon the site and extent of cardiac hypertrophy, HCM patients may develop left ventricular outflow obstruction, myocardial ischemia, mitral regurgitation or diastolic dysfunction [142]. Most, but not all, studies have shown a relationship between the degree of left ventricular hypertrophy and the risk for sudden cardiac death [143). Greater than 30mm of hypertrophy is one of the characteristics that identify a patient who may be a greater risk for sudden cardiac death [144], however, it is noteworthy that the risk relationship is linear [145] and appears to be more important for younger patients [146].

### Epidemiology

Hypertrophic cardiomyopathy in Africa was generally regarded as rare especially in the pre-echocardiographic era. We now know that HCM is common in all parts of the continent. In Ghana, an echocardiographic study showed that HCM was the 3^rd^ most common cause of cardiomyopathy after DCM and endomyocardial fibrosis [4]. In Ethiopia, an echocardiographically based study showed that HCM accounted for 34% of all cardiomyopathies [147]. In South Africa, HCM was present in 7% of patients with de novo HF [18]. 

The natural history of HCM is variable and many patients never exhibit any clinical manifestations. Mitral regurgitation is common in patients with left ventricular outflow tract obstruction and is usually caused by the distortion of the mitral valve apparatus from systolic anterior motion [144]. Progression of HCM to left ventricular dilatation with wall thinning occurs in about 3.5% of patients with higher rates (15%) being seen in specialized centers in developed country settings [148]. Most affected individuals will probably achieve a normal life expectancy without disability or the necessity for major therapeutic interventions [149]. On the other hand, in some patients, HCM is associated with profound complications, with the potential to result in disease progression or premature death [144, 149]. Patients at higher risk of sudden death include those with a history of resuscitation from sudden cardiac death, ventricular tachycardia on ambulatory monitoring, marked ventricular hypertrophy, syncope (especially in children), genetic mutations associated with an increased risk, an abnormal blood pressure response to exercise and a family history of sudden death [144]. 

### Clinical Characteristics

HCM may be newly diagnosed at any age and is usually suspected on the basis of the presence of a murmur or abnormal electrocardiogram. The physical examination in an asymptomatic patient with HCM may show a fourth heart sound or left ventricular lift. When there is significant outflow tract obstruction, a harsh crescendo-decrescendo systolic murmur develops that begins slightly after S1 and is heard best at the apex and left lower sternal border. Maneuvers that alter the degree of obstruction can cause a change in the murmur intensity.

### Diagnostic Considerations

Two-dimensional Doppler echocardiography is the diagnostic modality of choice. The classical finding is septal hypertrophy out of proportion to posterior wall hypertrophy but there is heterogeneity in the morphology of the septal hypertrophy [150]. Electrocardiography shows left ventricular hypertrophy and widespread, deep, broad T waves but has a different appearance when the hypertrophy is located mostly apically [144]. All first-degree relatives should undergo screening with echocardiography with earlier and more frequent screening for children, those engaged in competitive athletics or families where HCM follows a more malignant course [138]. Due to the unique genetic variants found in South Africa, patients with HCM referred for genetic testing are usually screened for the 3 most common variants before further extensive testing [139]. Such testing is recommended for first-degree relatives of HCM patients who do not have HCM or for patients with atypical presentations [144] but is not widely available in SSA.

### Therapy

Treatment goals for patients with HCM do not vary globally. Primary prevention of sudden cardiac death is best achieved with implantable cardiac defibrillators [151]. Beta-adrenergic blockers and verapamil are often used for rate control or HF symptoms when there is no outflow tract obstruction. Some clinicians prefer disopyramide over verapamil when there is significant outflow tract obstruction [149]. Patients whose symptoms do not improve with one drug may benefit from switching to another class but combined administration is not advantageous [152]. Surgical or catheter based myectomy is indicated for HF due to obstructive HCM that is refractory to drug therapy. Pure vasodilators, high-dose diuretics and positive ionotropes should generally be avoided as they may exacerbate left ventricular outflow obstruction.

## ENDOMYOCARDIAL FIBROSIS

### Description and Pathophysiology

Endomyocardial fibrosis (EMF) is a form of restrictive cardiomyopathy in which deposits of dense fibrous tissue in the endocardium impair ventricular diastolic function and distort the papillary muscles of the atrioventricular valves. This results in right, left or biventricular failure and atrioventricular valve regurgitation. Isolated left ventricular involvement is the rarest form in Africa [153]. The fibrotic process is usually located at the apex of the affected ventricle and extends to the inflow tract. 

### Epidemiology

The majority of cases have been reported in countries which are within 15 degrees of the equator [154] although there have also been sporadic cases in non-tropical countries [155-157]. The seminal knowledge regarding EMF comes from Uganda [7, 158] where up to 20% of patients referred for echocardiography are found to have the disease. The frequency of EMF cases has bimodal peaks around ages 10 and 30. Endomyocardial fibrosis affects Ugandan boys and girls equally, while adult women are affected twice as often as men [159]. Several factors have been associated with EMF in SSA. Eosinophilia is a major risk factor in SSA being inversely related to the length of time with the disease. Other important factors include ethnicity, diet, poverty, young age, female sex and infection. While in Uganda EMF is said to be more common among immigrants from neighboring Rwanda and Burundi [160], EMF also occurs in foreigners who have lived in endemic regions [161] suggesting that ethnicity is not a necessary causal factor. There are animal data to support the concept that prolonged ingestion of a diet rich in cassava and low in meats and fish results in a cardiomyopathy similar to EMF [162] as well as associations between infection with *Plasmodium *species [163] and *Schistosoma mansoni *(in Egypt) [164]. There is a paucity of genome based research in this area [165]. After over 6 decades of research, the cause remains elusive [166]. Prognosis is generally poor and death usually occurs within 2 years or slightly longer after diagnosis [165].

### Clinical Characteristics

The clinical features of EMF depend on the location and degree of cardiac involvement. A history of a febrile illness with or without urticaria is present in 30-50% of children and adolescents with EMF [167]. When right ventricular or tricuspid valve involvement predominates, lower extremity and abdominal swelling predominate. Left ventricular involvement may present with signs of restriction. End-stage EMF is notable for the presence of an exudative ascites with little or no peripheral edema [168]. Exudative pericardial effusion is also a common presentation. 

### Diagnostic Considerations

Most cases of EMF in the literature have been diagnosed by cardiac dilatation and apical calcification on chest x-ray and low voltage, atrioventricular block and other abnormalities on electrocardiogram [169]. Classic echocardiographic findings include intraventricular thrombus, pericardial effusion or atrioventricular valve restriction with regurgitation [159]. More recently, an echocardiographic screening study in Mozambique presented echocardiographic criteria for the diagnosis and staging of EMF [154]. The natural history of EMF using this staging criteria are yet to be described but will likely aid in determining the clinical significance of early stage EMF [154, 170].

### Therapy

Due to pathophysiology of eosinophilia, corticosteroids and immunosuppressive drugs are thought to be helpful in the early stages of the disease but there are no randomized clinical trials to support their use [171]. Diuretics are used symptomatically to relieve pulmonary and systemic venous congestion but caution is required as they may reduce ventricular filling pressures and cause symptoms of low output in the setting of restrictive physiology. Paracentesis may be required for symptomatic relief but recurrent ascites is the rule. In general, the response to medical therapy is dismal with 75% mortality at 2 years [172].

Where capabilities and expertise exist, EMF may be successfully treated with surgical endocardectomy and valve replacement. The operative mortality by may as high as 20% [173], however, surgical endocardectomy is the only therapy which offers a reasonable chance of long-term survival and good outcomes have been reported [156].

## ISCHEMIC HEART DISEASE

### Description and Pathophysiology

As a cause of HF in SSA, ischemic heart disease has always been considered a rarity until recently. As reported by Pobee and Biritwum, medical reports sent from Ghana (then the Gold Coast) to the Colonial Secretary in London showed that coronary heart disease was extremely uncommon from the 1890s to the mid 20^th^ century [174, 175]. In South Africa, however, there has long been a recognition of the increasing burden of ischemic heart disease noting a 12-fold increase in the prevalence of myocardial infarction at autopsy between 1959-1976 [176]. Further support for this transition comes from an autopsy based study in Kenya which showed that ischemic heart disease was the leading cause of cardiovascular death in a national referral hospital [177]. 

Clinical studies corroborate findings from necropsy studies. Alberts* et al.* demonstrated a high prevalence of traditional cardiovascular risk factors in a cross-sectional study in an impoverished area of South Africa [178]. Tobacco smoking and alcohol use was found in >50% of men in this study while obesity was highly prevalent (52%) among women. In this study, 32% of men and 19% of women had greater than 20% chance of a cardiovascular event in 10 years. In the Heart of Soweto study, modifiable risk factors for cardiovascular disease were common with 56% of patients being hypertensive, 44% obese and only 13% with no cardiovascular risk factors [11]. Reports from other African countries support these findings [179]. The Agincourt longitudinal study also showed a 65% increase in deaths due to ischemic heart disease, stroke and hypertensive heart disease between 1992 and 2005 [180]. 

The INTERHEART study demonstrated that the risk factors for ischemic heart diseases are common to all regions of the world; however, there are important differences in the distribution of these risk factors according to ethnic groups [181]. In sub-Saharan Africa, hypertension and diabetes were common risk factors among Black Africans while abdominal obesity, tobacco smoking and dyslipidemia were more common among European Africans [182]. Black Africans in the highest tertile of income had a higher risk of myocardial infarction than those in the lowest tertile of income. The relationship was reversed among European Africans and did not exist among colored Africans demonstrating important differences with regard to the epidemiologic transition of cardiovascular disease risk. 

### Epidemiology

Reports from SSA hospitals suggest that African patients with coronary disease are often younger than their Western counterparts. Other clinical and laboratory characteristics appear to be the same, as are the types of complications that occur [10, 183]. It is not clear that the natural history of ischemic cardiomyopathy is vastly different in SSA compared to other parts of the world. The relatively low prevalence of traditional cardiovascular risk factors is counterbalanced by rapid westernization and ethnic differences in risk factors suggesting that more intense study in this field is warranted [184].

### Clinical Characteristics

The few data concerning African patients with ischemic cardiomyopathy suggest that physical signs are few and a history of angina is uncommon [185]. Common complaints are fatigue and dyspnea [186]. Of all patients presenting with de novo presentations of HF, women are slightly more likely than men to complain of angina and are more likely to present with peripheral edema [18]. A female predominance of ischemic heart disease has also been found in Togo [187] but the opposite trend exists in South Africa [18] and Nigeria [188]. In a review of medical records of patients admitted to a medical ward with cardiovascular disease, investigators in Nigeria found that 22% presented with angina and 30% presented with ischemic cardiomyopathy [188]. The dominant clinical presentations of ischemic heart disease in Togo were stable angina (71%), silent ischemia (22%), myocardial infarction (5%) and unstable angina (2%) [187]. In the long-term, HF is a common manifestation of chronic coronary atherosclerosis developing in 44% of patients in Kenya [189]. Whether distinct presentations of ischemic heart disease are present or impact the ability to diagnose or treat appropriately is yet to be determined. 

### Diagnostic Considerations

The diagnosis of ischemic heart disease is usually based on a clinical picture and electrocardiographic findings. A high index of suspicion and broad differential, however, are necessary to perform an electrocardiogram in a patient with nonspecific signs or symptoms and this probably contributes to the low prevalence estimates [190]. Definitive diagnosis can be curtailed by the lack of high-technology diagnostic testing (i.e., nuclear or angiographic imaging) and the fact that enzyme testing is not available in many centers. In a Kenyan national referral hospital, confirmation of myocardial infarction was accomplished most commonly with the combination of electrocardiogram and cardiac enzymes (88%) and infrequently with angiography (8%) [189]. Echocardiography, by demonstrating regional wall motion abnormalities, has also been used when more advanced diagnostics are unavailable, however, this approach suffers from very low sensitivity (36-87%) [191]. 

### Therapy

Recommendations for the treatment of ischemic cardiomyopathy apply worldwide. An approach based on stage of disease emphasizes a goal-directed stepwise approach [192]. Incremental therapy including vasodilators, beta-adrenergic blockers, diuretics, digitalis, anti-arrhythmics and/or ionotropes should be used as indicated. A special issue for this population, however, is revascularization. Coronary angiography, when it is available, should be performed in patients presenting with HF who have angina or significant ischemia unless the patient is not eligible for revascularization of any kind. For patients with ischemic cardiomyopathy without severe symptoms who are eligible for coronary artery bypass grafting, one large international trial demonstrated no significant difference in all-cause mortality between medical therapy alone and medical therapy plus coronary artery bypass grafting [193]. There were significant differences in cardiovascular mortality and death or cardiovascular hospitalization favoring surgical revascularization [193]. For the SSA region where access to revascularization is limited, the focus should be on improving the quality of medical therapy for patients with ischemic cardiomyopathy.

## COR PULMONALE

### Description and Pathophysiology

Cor pulmonale was defined by the World Health Organization in 1963 as “hypertrophy of the right ventricle resulting from diseases affecting the function and/or structure of the lungs, except when these pulmonary alterations are the result of diseases that primarily affect the left side of the heart, as in congenital heart disease” [194]. Since then, the definition has expanded to include right ventricular hypertrophy, dilation, or both secondary to pulmonary hypertension which often leads to right HF [195]. Pulmonary hypertension is the sine qua non of cor pulmonale. Both anatomic (e.g., fibrosis or thromboembolic lesions) and functional factors (e.g., alveolar hypoxia or acidosis) contribute to an increased pulmonary vascular resistance. In the case of chronic hypoxemia, this state causes the release or suppression of mediators responsible for vascular tone and vascular cell proliferation resulting in alteration in vascular function, vessel remodeling and onset of persistent pulmonary hypertension that is refractory to correction with supplemental oxygen. Pulmonary hypertension increases the work of the right ventricle which leads to right ventricular enlargement associated with dilatation and hypertrophy. The interval between the onset of pulmonary hypertension and right HF is unknown but it is likely related to the etiology of pulmonary hypertension. Lung damage from tuberculosis infection contributes to many of the cases of cor pulmonale in SSA [104]. Other important causes of pulmonary hypertension and right HF which are likely to contribute most to morbidity in SSA are HIV, hemoglobinopathies, chronic obstructive pulmonary disease, interstitial lung disease, high altitude and chronic thromboembolic disease [196].

### Epidemiology

Available data suggest that 20 to 25 million people in low- and middle-income countries have some form of pulmonary vascular disease, representing >97% of the global burden [197]. Right HF appears to be one of the leading types of HF in South Africa (27% of 844 de novo cases) [18]. This is in contrast to somewhat lower prevalence estimates (4-17%) from older studies [3]. In Zimbabwe and Ethiopia, where schistosomiasis is endemic, pulmonary hypertension and right HF are commonly seen [198]. HIV-related pulmonary hypertension was found in 8% of de novo cases of HF in the Heart of Soweto study and was more common among young women [199]. In Burkina Faso and Zimbabwe, the prevalence rates are 0.6% and 6%, respectively [135, 200]. In a study of 128 patients with pulmonary embolism in Kenya, chronic thromboembolic pulmonary hypertension or symptomatic HF developed in 30 (23%) patients [201]. The impact of household air pollution on cardiovascular disease is gaining international attention, however, the effect on pulmonary hypertension and right HF has not been systematically studied. Pilot data from Kenya, however, suggest a relationship between right HF, reduced cook hut ventilation, pulmonary airflow obstruction and functional limitations [202]. 

### Clinical Characteristics

Clinical manifestations of pulmonary hypertension and right HF are often overshadowed by the signs and symptoms of the underlying causative condition. There is no history specific for pulmonary hypertension but the history should lead to clues about the cause (e.g., recurrent pain crises in someone with sickle cell disease in contrast with a history of treatment for pulmonary tuberculosis). The most sensitive sign of long-standing pulmonary hypertension is an accentuated component of S2 which may also be palpable in the pulmonic area. A right ventricular lift may be seen. With very high pulmonary artery pressures, diastolic and systolic murmurs of pulmonic and tricuspid regurgitation may be heard along with a systolic ejection sound and right ventricular S3 gallop. In severe right HF, cardiac enlargement, distended neck veins, hepatomegaly, ascites and peripheral edema are present. Often, the degree of ascites may be out of proportion to the degree of peripheral edema. Small series show that cyanosis is also common [198]. The utility of findings of electrocardiogram, CXR and echocardiography probably follow patterns similar to other regions of the world.

### Diagnostic Considerations

Because of the diversity of diseases that cause pulmonary hypertension and right HF, no single strategy of work-up exists. Instead, historical and symptomatic clues should lead the clinician down the appropriate diagnostic pathway. When lung parenchymal or airway disease is present, pulmonary function tests will frequently reveal the nature and degree of impairment. Transbronchial biopsy, bronchoalveolar lavage and open lung biopsy are diagnostic options in patients with interstitial lung disease. Patients with cryptogenic pulmonary hypertension should undergo testing to detect pulmonary arterial emboli or other causes of obstruction with perfusion radionuclide testing or computed tomography scanning depending on availability. Serology for hepatitis B surface antigen, antinuclear antibody, rheumatoid factor and cryoglobulins may confirm a clinical suspicion for pulmonary vasculitis.

Right heart catheterization is the gold standard for the diagnosis of pulmonary hypertension. However, echocardiography is frequently used to screen, diagnose and characterize pulmonary hypertension and right HF in many settings owing to the relative affordability and accessibility of echocardiography and that fact that it is non-invasive. The overall correlation between echocardiographically determined and invasively measured pulmonary systolic pressure is good (r=0.7) with 83% sensitivity and 72% specificity according to a meta-analysis of the literature [203]. Single center studies have also shown that echocardiography underestimates pulmonary artery systolic pressure more so than overestimates it, but can do both [204]. 

### Therapy

The underlying disease is the focus of therapy and is the best way to reduce ventricular pressure work. If right HF has not occurred, a major goal is to prevent this complication as it carries a high mortality (70% within 6 months) [205]. Relief of hypoxia is of prime importance in reducing pulmonary hypertension although some patients may have irreversible changes with alterations in the pulmonary vascular bed. Caution should be used when the partial pressure of carbon dioxide is high due to the threat of respiratory acidosis. 

Diuretics should be used to diminish hepatic and peripheral edema [206] but caution is advised when administering to avoid decreasing the cardiac output. The evidence to support the use of anticoagulation therapy is mostly observational and supports its use in cases of patients with idiopathic pulmonary hypertension [207] but is also generally recommended for patients with pulmonary hypertension due to chronic thromboembolic disease, heredity or adverse effects of medication. A small study (n=15) showed that digitalis improves right ventricular ejection fraction in patients with pulmonary hypertension and biventricular failure due to chronic obstructive pulmonary disease [208]. However, these patients may also be more sensitive to the effects of digitalis and require close monitoring to prevent adverse effects. Recommendations for the use of advanced therapies (i.e., treatment directed at pulmonary hypertension itself rather than the cause of the pulmonary hypertension) restrict the use of vasodilators to subgroups of patients where the risk/benefit ratio is low and also require demonstration on invasive testing that vasoreactivity exists before initiation [206]. When indicated, dihydropyridine calcium channel blockers or diltiazem are the first line agents.

## OTHER FORMS OF HF

Lesser common forms of HF in SSA include high-output HF due to thiamine deficiency and arrhythmogenic right ventricular cardiomyopathy (ARVC). Thiamine (vitamin B1) plays an important role in carbohydrate metabolism [209]. Thiamine is not produced endogenously and is stored in the body in small amounts. Therefore, adequate intake or supplementation is necessary to avoid deficiency [210]. The HF of thiamine deficiency (so called wet beri-beri) is a chronic disease characterized by a peripherally vasodilated state that leads to fluid retention through activation of the renin-angiotensin-aldosterone system. The clinical effect is high-output heart failure. Thiamine deficiency is less common than in years past but have been noted to account for up to 32% of cases of HF in one South African center [211]. There is also a historic overlap between chronic alcoholism and thiamine deficiency where the HF associated with alcoholism was once attributed solely to nutritional deficiencies. When thiamine deficiency is clearly established as the cause of HF, replacement therapy may improve diuresis and ejection fraction though results are mixed [212, 213].

ARVC is a heart muscle disease characterized by HF, ventricular arrhythmias and sudden cardiac death. The hallmark of ARVC is fibro-fatty replacement of the myocardium (mostly the right ventricle) which predisposes to arrhythmias. ARVC has genetic determinants related to mutations in genes that encode proteins of the cardiac desmosomes [214]. The first cases from Africa were reported in 2000 [215] and subsequent studies have demonstrated that mutations in the major gene that is implicated in ARVC worldwide (plakophylin- 2) are also common in SSA [216]. Due to limited availability of advanced electrophysiology expertise and diagnostics, there is a dearth of reports of ARVC outside of South Africa. The ARVC Registry of South Africa reported that the clinical features of ARVC in South Africa were similar to the rest of the world and that most patients with ARVC were white [216]. The overall mortality rate from ARVC is similar to the general South Africa population.

## CONTEMPORARY TREATMENT PATTERNS

The use of evidence-based therapies for HF has historically been low in SSA. Adewole *et al*., for example, demonstrated that while the use of angiotensin converting enzyme inhibitors for patients with HF has increased from 37% in 1992 to 65% in 1994 the proportion of patients prescribed this class of medication remains strikingly low [14]. In Cameroon, only 20% with HF were prescribed beta-blockers from 1998-2001 [13]. 

The THESUS-HF study demonstrated that the most commonly prescribed intravenous medication for acute decompensated HF in SSA is furosemide [6]. In addition, angiotensin converting enzyme inhibitor or angiotensin receptor blocker medications are prescribed in about 80% of patients at discharge, 1-month or 6-monts of follow-up. Beta-blocker use is strikingly low being prescribed in approximately 30% of patients at discharge and less than 50% at 6-months of follow-up. The proportion of patients using beta-blockers in the discharge and immediately post-discharge period in SSA (~30%) is lower than that of digoxin (~60%), despite that international guidelines recommend digoxin as a fourth-line agent or for use in conjunction with a beta-blocker for patients with advanced HF [74, 217]. The reasons for this prescribing pattern is unclear but may be related some of the older controversies regarding beta-blockers and African-American patients [218-220]. Lastly, the combination of hydralazine and nitrates in addition to standard therapy for HF (angiotensin converting enzyme inhibitors, angiotensin receptor blockers, beta-blockers for at least three months, digoxin, spironolactone, and diuretics) has been shown to be effective in patients of African descent with HF [221] but were used in <10% of patients in THESUS-HF [6].

## FUTURE DIRECTIONS

There have been many recent advances in knowledge of HF from around the world [222]. While many of these findings will apply to Africans with HF, there are reasons to specifically study the syndrome of HF in the region including understanding the population-specific disease burden, the vast genetic heterogeneity in SSA, geographic, social and cultural diversity and secular trends that may impact risk factors for various forms of HF [223]. 

Scientific approaches to gain a better understanding of the epidemiology, treatment patterns and outcomes of HF include registries, population-based cohort studies and clinical trials. Some examples of these already exist on the continent [6, 18]. However, more research is needed in some areas. As an example, despite recognition of cor pulmonale as a significant contributor to HF in SSA, the causes remain elusive and probably relate to unique exposures in SSA [e.g., household air pollution, dust from coal mining, high altitude) [197, 224]. Knowing the socio-cultural context of the disease allows researchers in SSA to ask the most appropriate questions using the most appropriate methods. As Fonn suggests, research conceptualized, conducted, analyzed and published by Africans is crucial for Africa to meet the health needs of her people [225]. As echocardiographic technology advances and devices become more portable, we are also poised to diagnose and understand HF during its earliest manifestations (e.g., myocardial strain imaging) [226] and in the community (e.g., portable hand-held devices) [227]. Clinical research should be coupled with investigations into the biologic basis for various forms of HF. For example, increasing access to echocardiography and early diagnosis may foster a better understanding of EMF while exploration of molecular mechanisms may uncover therapeutic targets [171]. Lastly, where the causes and treatments for some conditions are well known (e.g., RHD, hypertensive heart disease), these conditions still contribute to significant morbidity and mortality on the continent. Research into best strategies to implement best practices for prevention and treatment of HF in SSA due to these conditions are also sorely needed [228].

## CONCLUSION

The syndrome of HF remains a major public health issue for many countries in SSA. Systolic HF appears to dominate. While ischemic heart disease is the predominate in high-income countries, the most common etiologies in SSA are distinct with hypertension, valvular heart disease and non-ischemic cardiomyopathies being the most commonly reported forms. This trend has generally been preserved over the last 4 decades; however, larger contemporary studies highlight the emergence of right-sided HF and ischemic heart disease and the waning importance of infectious causes. While atherosclerotic heart disease is still a relatively rare cause of HF, specific investigation for atherosclerotic heart disease using contemporary means has only been performed in a handful of studies. Current treatment patterns highlight the inappropriately low usage of proven therapies for HF. With the recent growth in scientific and public interest in HF, clinicians in SSA stand to gain important knowledge and a network in which to study treatment and outcomes that are relevant to the region and impactful worldwide.

## Figures and Tables

**Table 1.** Etiology of Heart Failure in Sub-Saharan Africa by Decade

	Pre-1960	1960-1969	1970-1979	1980-1989	1990-1999	2000-present
**Number of studies**	2	4	3	1	4	11
**References**	[47, 48]	[7, 49-51]	[52-54]	[55]	[56-59]	[4, 6, 13, 18, 60-66]
**Total number of patients**	464	2546	872	315	1669	4298
**Etiology**						
Valvular disease	24.8%	22.4%	12.7%	13.7%	21.0%	16.9%
Hypertension	18.1%	24.7%	38.3%	11.7%	15.0%	38.0%
Cardiomyopathy	28.4%	10.6%	25.5%	47.0%	26.4%	26.0%
Cor pulmonale	8.0%	8.6%	5.7%	6.3%	3.9%	1.5%
Pericardial disease	5.2%	3.2%	5.2%	0.0%	16.8%	2.8%
Ischemic heart disease	0.2%	0.6%	0.3%	0.0%	1.1%	5.3%
Endomyocardial fibrosis	0.0%	3.8%	0.0%	0.0%	0.0%	1.0%
Syphilitic heart disease	4.7%	10.8%	1.3%	0.0%	0.0%	0.0%
Congenital	4.3%	2.2%	0.7%	0.0%	0.1%	1.8%
Other/Unknown	6.3%	13.0%	10.3%	21.3%	15.7%	8.3%
